# When roots talk to shoots about flooding

**DOI:** 10.1093/plphys/kiad464

**Published:** 2023-08-22

**Authors:** José Manuel Ugalde, Amanda A Cardoso

**Affiliations:** Assistant Features Editor, Plant Physiology, American Society of Plant Biologists; Institute of Crop Science and Resource Conservation (INRES)—Chemical Signalling, University of Bonn, 53113 Bonn, Germany; Assistant Features Editor, Plant Physiology, American Society of Plant Biologists; Department of Crop and Soil Sciences, North Carolina State University, Raleigh, NC, 27695, USA

Global warming is associated with major changes in rainfall patterns and thus with increases in water extreme events, such as drought and flooding (IPCC 2022). Both extremes are detrimental for land plants, hindering their growth and ultimately causing premature death. A critical distinction between drought and flooding lies in the timeframe of their occurrence. Whereas droughts can take weeks to establish in the field, flooding can rapidly occur (within hours to days) following a sudden downpour, especially in areas of plains with poor soil drainage (Voesenek and Bailey-Serres 2015). Therefore, plants have considerably less time to activate mechanisms to tolerate flooding than to tolerate drought.

During flooding, plants can be subjected to waterlogging (i.e. when only the roots remain under water) or to submergence (i.e. when the entire plant is covered by water) (Sasidharan et al. 2017). When waterlogged, the roots face low levels of oxygen in the soil, which results in wide metabolic alterations in this organ. Alterations include inhibition of mitochondrial respiration and activation of fermentation pathways, activation of Ca^2+^ signaling, increased levels of reactive oxygen species (ROS) and lipid peroxidation, protein degradation, and alterations in hormones (Bailey-Serres and Voesenek 2008; Voesenek and Bailey-Serres 2015; Da-Silva and do Amarante 2020). Signaling mediated by Ca^2+^ and ROS in roots constitutes a critical player triggering tolerance mechanisms against hypoxia, including transcript accumulation and aerenchyma formation (Liu et al. 2017; Voesenek and Bailey-Serres 2015). Even though roots are the primary organs directly affected during waterlogging, the shoot also experiences metabolic and physiological alterations. Leaves, for instance, accumulate ROS after only a few hours following waterlogging (Da-Silva and do Amarante 2020). Gene expression is also substantially altered in leaves when roots are under water (Hsu et al. 2011). Therefore, a systemic signaling likely propagates from roots to the shoot during waterlogging, which might be essential for plant acclimation and survival.

In this issue of *Plant Physiology*, Pelaéz-Vico et al. (2023) shed new light on the rapid processes that occur during the early onset of waterlogging. The authors tested the hypothesis that the systemic response of plants exposed to waterlogging involves a ROS wave, which was previously reported to depend on RBOHD, GLR3.3/3.6, and PIP2;1. To measure waterlogging-dependent variations in ROS and Ca^2+^ levels in leaves, the authors fumigated Arabidopsis (*Arabidopsis thaliana*) with fluorescent dyes for ROS (H2DCFDA) or Ca^2+^ (Fluo-4-AM) before exposing the plants to waterlogging ranging for up to 60 min. The fluorescence of these dyes was monitored in the complete aerial part using a Lumina system. Their results indicated that Ca^2+^ levels in leaves increased after only 10 min of root waterlogging followed by ROS, which showed to increase only after 20 min. Furthermore, by using chemical inhibitors and mutant lines, the authors showed that this systemic waterlogging-induced ROS signal depends on RBOHD, GLR3.3/GLR3.6, and PIP2; 1–2, which are the same key components of the systemic ROS wave under other types of stress.

Because the systemic response to waterlogging was surprisingly fast, the authors investigated gene expression changes in the early stages of waterlogging (10, 30, and 60 min) via RNA-seq. Most of the genes whose expression was altered by waterlogging correspond to genes previously reported to be related to hypoxia or submergence responses (Bui et al. 2020; Tamura and Bono 2022). Because waterlogging promotes systemic changes in signaling molecules such as ROS and Ca^2+^ and promotes a change in the transcriptomic profile of the plant, the authors tested whether a short waterlogging pretreatment of 60 min would confer resistance to a posterior submergence event. Waterlogged plants showed a lower percentage of leaf injuries after recovery compared with non-waterlogged plants, indicating that waterlogging can efficiently prepare plants to better cope with a later submergence event (Fig. 1).

**Figure 1.**
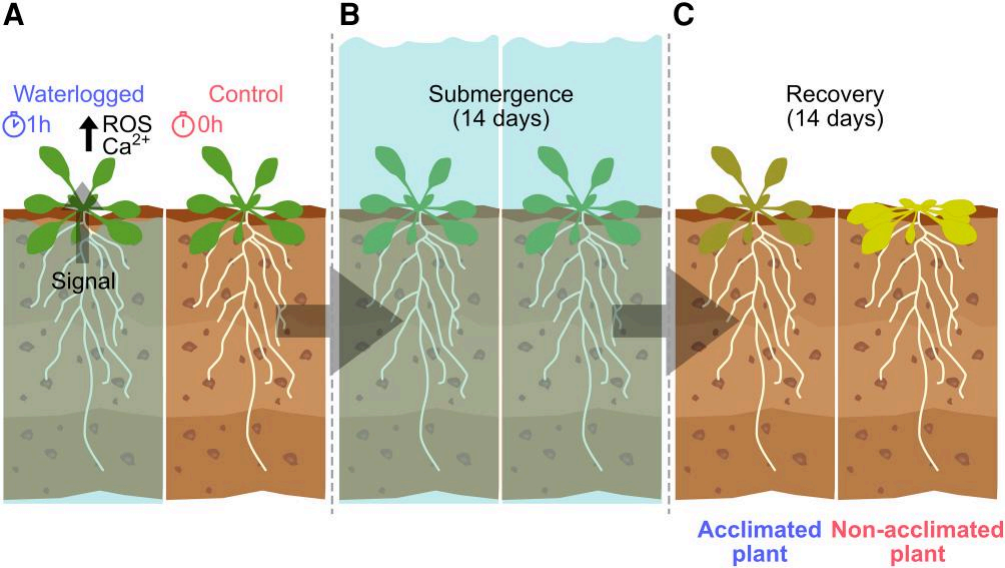
Root waterlogging promotes submergence stress tolerance by acclimating plants. **A)** Waterlogging treatments promote increased Ca^2+^ and ROS levels in leaves. **B–C)** One-hour root waterlogged roots or control plants, which were not exposed to waterlogging, were exposed to 14 days of submergence (**B**) followed by 14 days of recovery (**C**). Plants that were initially waterlogged showed to be acclimated, hence more tolerant to submergence. Adapted from Peláez-Vico et al. (2023). Figure created by J.M.U. in Affinity Designer (Version 2.1.0).

The study of Pelaéz-Vico et al. (2023) shows how a waterlogging-triggered systemic response primes plants for flooding adaptation before it escalates, highlighting the importance of root-shoot communication. Surprisingly, the absence of upregulated ethylene signaling genes indicates that other mechanisms, such as mechanoreceptors or hydraulic changes, might precede ethylene signaling during early waterlogging. One of the most relevant findings suggests that the waterlogging-induced ROS wave is part of a versatile molecular pathway that responds similarly to other stresses such as wounding or excess light. Further questions, however, remain to be explored, including evaluating the effects of gradual water rising instead of abrupt flooding mimicking a pouring rain or testing the dynamic changes of ROS and Ca^2+^ in crops with varying sensitivity to low oxygen conditions such as tomato plants. Ethylene pretreatments on this setup could give a deeper insight into the potential interactions between ethylene signaling and the early ROS wave during waterlogging. Measurements can include mutants for the key components of ethylene signaling (e.g. *etr1*) or proteasome-mediated signaling on hypoxia (e.g. PGBs, ATRs). Lastly, understanding ROS dynamics in pre-adapted plants would provide valuable information on how prior conditioning the subsequent response affects waterlogging events. This research opens up exciting possibilities for future studies, advancing our understanding of plant responses to waterlogging stress and the intricate signaling networks that occur when roots talk to shoots.

## Dive Curated Terms

The following phenotypic, genotypic, and functional terms are of significance to the work described in this paper:

ETR1 Gramene: AT1G66340
ETR1 Araport: AT1G66340

ethylene Gramene: Hormone
ethylene Araport: Hormone

root AmiGo: PO:0009005

RBOHD Gramene: At5g47910
RBOHD Araport: At5g47910
